# *Lactobacillus reuteri* CCFM8631 Alleviates Hypercholesterolaemia Caused by the Paigen Atherogenic Diet by Regulating the Gut Microbiota

**DOI:** 10.3390/nu14061272

**Published:** 2022-03-17

**Authors:** Qianqian Wang, Yufeng He, Xiu Li, Ting Zhang, Ming Liang, Gang Wang, Jianxin Zhao, Hao Zhang, Wei Chen

**Affiliations:** 1 State Key Laboratory of Food Science and Technology, Jiangnan University, Wuxi 214122, China; 7160112022@stu.jiangnan.edu.cn (Q.W.); 7170112055@stu.jiangnan.edu.cn (Y.H.); lixiu@jiangnan.edu.cn (X.L.); wanggang@jiangnan.edu.cn (G.W.); zhanghao61@jiangnan.edu.cn (H.Z.); chenwei66@jiangnan.edu.cn (W.C.); 2 School of Food Science and Technology, Jiangnan University, Wuxi 214122, China; 3 Infinitus (China) Company Ltd., Guangzhou 510645, China; tinca.zhang@infinitus-int.com; 4 (Yangzhou) Institute of Food Biotechnology, Jiangnan University, Yangzhou 225004, China; 5 National Engineering Research Center for Functional Food, Jiangnan University, Wuxi 214122, China; 6 Wuxi Translational Medicine Research Center, Jiangsu Translational Medicine Research Institute Wuxi Branch, Wuxi 214122, China

**Keywords:** atherosclerosis, gut microbiota, probiotics, short chain fatty acids, hypercholesterolaemia

## Abstract

Cardiovascular disease has one of the highest global incidences and mortality rates. Atherosclerosis is the main cause of cardiovascular disease, and hypercholesterolaemia and hyperlipidaemia are the main risk factors for the development of atherosclerosis. Decreasing serum cholesterol and triglyceride concentrations is considered to be an effective strategy to prevent atherosclerotic cardiovascular disease. Previous studies have shown that many diseases are related to gut microbiota dysbiosis. The positive regulation of the gut microbiota by probiotics may prevent or treat certain diseases. In this study, *Lactobacillus reuteri* CCFM8631 treatment was shown to decrease plasma total cholesterol (TC), low-density lipoprotein–cholesterol, aspartate transaminase, alanine transaminase and trimethylamine N-oxide concentrations, decrease liver TC and malondialdehyde concentrations and recover liver superoxide dismutase concentrations in mice fed a Paigen atherogenic diet. In addition, *L. reuteri* increased the faecal short-chain fatty acid content (acetate, propionate and butyrate), which was accompanied by an increase in the relative abundance of faecal Deferribacteres, *Lachnospiraceae* NK4A136 group, *Lactobacillus* and *Dubosiella*; a decrease in the relative abundance of *Erysipelatoclostridium* and *Romboutsia* and the activation of butanoate and vitamin B6 metabolism, leading to the alleviation of hypercholesterolaemia.

## 1. Introduction

Cardiovascular disease (CVD) has one of the highest global incidences and mortality rates. Atherosclerosis is the main cause of CVD, and hypercholesterolaemia and hyperlipidaemia are the main risk factors for the development of atherosclerosis [1]. The World Health Organization (WHO) has predicted that by 2030, the mortality rate of cardiovascular-related diseases will reach 40%, accounting for approximately 23.6 million deaths globally [2]. Clinical studies have shown that the risk of heart disease is three times higher in people with hypercholesterolaemia than in those with normal blood lipid profiles. The incidence rate of CVD increases by 2–3% when serum cholesterol concentrations increase by 1% [3]. Hypercholesterolaemia and hyperlipidaemia are characterised by high total cholesterol (TC), low-density lipoprotein (LDL) and triglyceride (TG) concentrations and low high-density lipoprotein (HDL) concentrations in serum [4]. High serum cholesterol and TG concentrations are the main causes of not only atherosclerosis and atherosclerosis-related diseases [5] but also non-alcoholic fatty liver disease [6].

Decreasing serum cholesterol and TG concentrations is considered to be an effective method to prevent atherosclerotic CVDs. To achieve this goal, statins are widely used for the treatment of hypercholesterolaemia and hyperlipidaemia to prevent CVD [7]. Rosuvastatin is one of the most effective statins currently in use. However, while these drugs reduce blood lipid and cholesterol concentrations, they have side effects, such as abnormal liver enzyme concentrations, rhabdomyolysis and an increased risk of liver and muscle injury [8]. Therefore, it is becoming increasingly common to explore non-drug interventions, such as dietary interventions, including reducing the intake of saturated fat, increasing the intake of monounsaturated and polyunsaturated fatty acids and taking probiotic supplementation, to reduce blood lipid and cholesterol concentrations.

Probiotics are living microorganisms recognised by the Food and Agriculture Organization of the United Nations and the WHO for their ability to produce beneficial effects on human health [9], and they are also considered to have beneficial effects on animal health. The cholesterol-lowering effect of probiotics has attracted much research attention. In clinical and animal experiments, *Lactobacillus* and *Bifidobacterium* have been found to have good cholesterol-lowering potential. *Lactobacillus* reduces cholesterol through various possible mechanisms [10,11,12], such as reducing cholesterol synthesis by inhibiting the enzyme 3-hydroxy-3-methyl-glutaryl-coenzyme A reductase; producing bile salt hydrolase to catalyse the hydrolysis of conjugated bile acids into unconjugated bile acids, thus promoting the elimination of bile acids and the catabolism of cholesterol; increasing the expression levels of LDL-receptor in the liver, which results in the removal of LDL-cholesterol (LDL-C) from the blood, thus reducing the TC concentration; absorbing and assimilating cholesterol; producing organic acids and reducing plasma TC and LDL-C concentrations. Thus, probiotics are natural, safe non-drug supplements that reduce serum cholesterol and TG concentrations and consequently prevent CVD. In this study, different probiotics were evaluated for their regulatory effects on hyperlipidaemia and their effects on the intestinal flora and intestinal metabolites in mice fed a Paigen atherogenic diet.

## 2. Materials and Methods

### 2.1. Probiotic Strains and Culture

Three strains of *Lactobacillus* and two strains of *Bifidobacterium* were isolated from healthy human faeces (Table 1). This study did not involve human experiments. The collection of faecal samples had no foreseeable risk of harm or discomfort to the participants. Faecal samples were collected from healthy humans for public health research purposes, and the participants who volunteered their faecal samples or their legal guardians provided written informed consent.

### 2.2. Mice and Dietary Interventions

Sixty female C57BL/6J mice aged 5 weeks and weighing 16–18 g were purchased from the Shanghai Slac Laboratory Animal Co., Ltd. (Shanghai, China). All experiments were carried out in the Experimental Animal Center of Jiangnan University. Mice were housed in clean individually ventilated cages at 25 ± 2 °C and 50 ± 5% humidity under a 12-h/12-h light–dark cycle. The mice had free access to food and water during the experimental period. All animal experiments were reviewed and approved by the Ethics Committee of Jiangnan University. JN. No 20180415c0901007(62). The control diet (No.: TP23302) and the Paigen atherogenic diet [13] (No.: TP28600; 15% fat, 1.25% cholesterol and 0.5% cholate) were purchased from TROPHIC Animal Feed High-Tech Co., Ltd. (Nantong, China) and stored at 4 °C.

After 1 week of acclimatisation, the mice were randomly divided into seven groups (*n* = 8–10 per group) and assigned diets according to the following scheme for 16 weeks: (1) the control group received a control diet plus 0.2 mL saline; (2) the Paigen group received a Paigen atherogenic diet plus 0.2 mL saline; (3) the FCQAS2M1 group received a Paigen atherogenic diet plus 0.2 mL (1 × 10^9^ colony-forming units [CFU]) of *Lactobacillus plantarum* FCQAS2M1; (4) the CCFM8631 group received a Paigen atherogenic diet plus 0.2 mL (1 × 10^9^ CFU) of *L. reuteri* CCFM8631; (5) the FSDQZ6M1 group received a Paigen atherogenic diet plus 0.2 mL (1 × 10^9^ CFU) of *L. casei* FSDQZ6M1; (6) the FSXAB2M1 group received with a Paigen atherogenic diet plus 0.2 mL (1 × 10^9^ CFU) of *Bifidobacterium breve* FSXAB2M1 and (7) the FZJZD9M1 group received a Paigen atherogenic diet plus 0.2 mL (1 × 10^9^ CFU) of *B. adolescentis* FZJZD9M1. Saline and bacteria suspensions were administered by gavage once daily for 16 weeks. Food and water intake and body weight were recorded once per week. Two more mice were set in the control group and Paigen group respectively, which were used to assess progress in animal model construction. Histopathological staining for these four mice was carried out during the experiment to determine the end time of the experiment.

*Lactobacillus* and *Bifidobacterium* strains were activated for three generations at 3% inoculum (*v*/*v*) in MRS medium containing 0.05% cysteine. After the strain was activated, it was further cultivated in large quantities. *Lactobacillus* and *Bifidobacterium* were collected by centrifugation at 6000× *g* for 15 min at 4 °C. The collected bacteria were washed three times with pre-cooled sterile normal saline (containing 0.05% cysteine) and resuspended in a small volume of pre-cooled 30% sucrose solution and stored at −80 °C. At the same time, the amount of bacteria in the resuspension was evaluated by the plate colony technique. Before it was used for gavage, in order to eliminate the influence of residual sucrose on the bacterial suspension, the stains were washed three times with pre-cooled sterile normal saline (containing 0.05% cysteine), and then diluted with sterile normal saline to a viable count of 1 × 10^9^ CFU/mL. Mice in the control group were treated with sterile saline without lactobacteria and bifidobacteria.

### 2.3. Sample Collection

Mouse faeces were collected at 16 weeks and stored immediately at −80 °C. At the end of the experiment, the mice were fasted overnight, anaesthetised by intraperitoneal injection of 1% pentobarbital sodium at 100 mg/kg body weight and then sacrificed. Blood samples were collected in anticoagulant tubes containing dipotassium ethylenediaminetetraacetic acid, and plasma was obtained by centrifugation at 3000× *g* for 20 min at 4 °C. Liver tissues were removed, weighed and snap-frozen in liquid nitrogen. Plasma samples and liver tissues were stored at −80 °C. The fixed end of the largest lobe of the liver and the aorta (containing the aortic arch and the abdominal aorta) were stored in paraformaldehyde.

### 2.4. Biochemical Analyses

Plasma and hepatic TC and TG concentrations; plasma HDL-cholesterol (HDL-C), LDL-C, aspartate transaminase (AST) and alanine transaminase (ALT) concentrations; hepatic superoxide dismutase (SOD) activity and hepatic MDA concentration were measured using commercial assay kits purchased from Jiancheng Bioengineering Institute (Nanjing, China). The levels of TNF-α, IL-1β and IL-6 in the liver were quantified by an enzyme-linked immunosorbent assay (ELISA) kit (R&D Systems, Minneapolis, MN, USA). Plasma trimethylamine N-oxide (TMAO) concentration was determined by liquid chromatography with tandem mass spectrometry, as previously described [14]. Liver index values were calculated as liver weight (g)/body weight (g).

### 2.5. Histopathological Analysis

Tissue staining was performed according to a previously reported method [15]. The liver, the abdominal aorta and the aortic arch were fixed in 4% paraformaldehyde for haematoxylin and eosin (HE) staining and Oil Red O staining. Histological injury of liver was evaluated independently by two blinded pathologists according to the SAF scoring system from three aspects including the number of medium- and large-sized intracytoplasmic lipid droplets, the degree of lobular inflammation and the extent of hepatocyte ballooning measured [16]. Medium- and large-sized intracytoplasmic lipid droplets <5%:0, 5–33%:1, 34–66%:2, >66%:3; lobular inflammation (count of necrotic lesions on 20× microscope): none:0, <2:1, 2–4:2, >4:3; hepatocyte ballooning: none:0, few:1, many:2. The area of lipid droplets was quantified by the Image-Pro Plus (Version 6.0, Media Cybernetics, Rockville, MD, USA).

### 2.6. Faecal Short-Chain Fatty Acid Analysis

Faecal short-chain fatty acid (SCFA) concentrations were determined using a modification of a previously described method [17].

### 2.7. Analysis of the Gut Microbiota in Faecal Samples

The microbial community composition in faecal samples was determined according to a previously described method [18].

### 2.8. Statistical Analysis

Data are reported as the mean ± standard deviation with statistical significance inferred when *p* < 0.05. Most parameters were analysed by a one-way analysis of variance, with a post hoc least significant difference test, using SPSS software (version 22.0; IBM, Armonk, NY, USA) or GraphPad Prism (v6.0; GraphPad Software, La Jolla, CA, USA).

## 3. Results

### 3.1. Lactobacillus Reuteri CCFM8631 Has the Potential to Reduce Blood Lipid Concentrations and Alleviate Liver Injury after Paigen Atherogenic Diet Treatment

**Figure 1:**
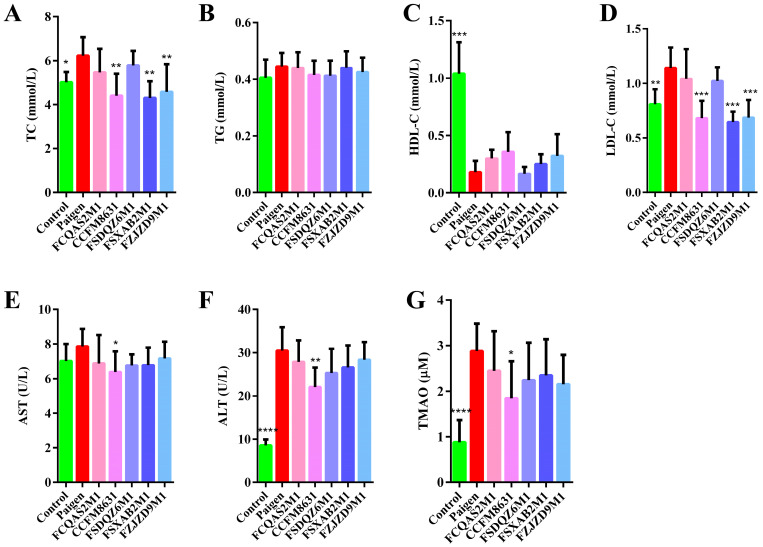
Analysis results of plasma biochemical parameters. (**A**) TC level. (**B**) TG level. (**C**) HDL-C level. (**D**) LDL-C level. (**E**) AST level. (**F**) ALT level. (**G**) TMAO level. *n* = 8. Compared with Paigen group, * *p* < 0.05, ** *p* < 0.01, *** *p* < 0.001, **** *p* < 0.0001.

With the extension of feeding time, the body weight of mice gradually increased. From the fifth week, the body weight of mice in Paigen group were significantly higher than those in the control group. *Lactobacillus* and *Bifidobacterium* supplementation tended to reduce the body weight of mice. *Lactobacillus* and *Bifidobacterium* supplementation had no significant effects on food intake and water intake (Figure S1). As shown in Figure 1A–D, the blood lipid profile significantly differed between the control mice and mice fed a Paigen atherogenic diet. TC and LDL-C concentrations significantly increased and HDL-C concentrations significantly decreased, but TG concentrations did not change significantly after mice were fed a Paigen atherogenic diet. The probiotic interventions appeared to alleviate the dyslipidaemia caused by the Paigen atherogenic diet. *L. reuteri* CCFM8631, *B. breve* FSXAB2M1 and *B. adolescentis* FZJZD9M1 significantly decreased TC and LDL-C concentrations in mice fed a Paigen atherogenic diet. Although *L. plantarum* FCQAS2M1 and *L. casei* FSDQZ6M1 decreased TC and LDL-C concentrations to some extent, these effects were not statistically significant. Probiotic supplementation also had a minor, but nonsignificant, effect on TG and HDL-C concentrations. AST concentration is an important indicator of hepatocyte necrosis. As shown in Figure 1E, there was a slight difference in AST concentrations between the control and Paigen groups. Probiotic supplementation decreased plasma AST concentrations, with *L. reuteri* CCFM8631 showing the most significant effect. ALT concentration is an important parameter that reflects liver disease. As shown in Figure 1F, there was a highly significant difference in ALT concentrations between the control and Paigen groups, indicating that the livers of mice fed a Paigen atherogenic diet may be seriously damaged. Probiotic supplementation decreased plasma ALT concentration and appeared to alleviate the liver injury caused by a Paigen atherogenic diet. In particular, *L. reuteri* CCFM8631 had a significant alleviating effect on Paigen atherogenic diet-induced liver damage. As shown in Figure 1G, TMAO concentration was significantly higher in the Paigen group than in the control group. Probiotic supplementation slightly reduced plasma TMAO concentrations, with *L. reuteri* CCFM8631 showing a significant effect.

### 3.2. Lactobacillus Reuteri CCFM8631 Reduced the Lipid Cholesterol Concentration and Oxidative Liver Damage Caused by a Paigen Atherogenic Diet

**Figure 2:**
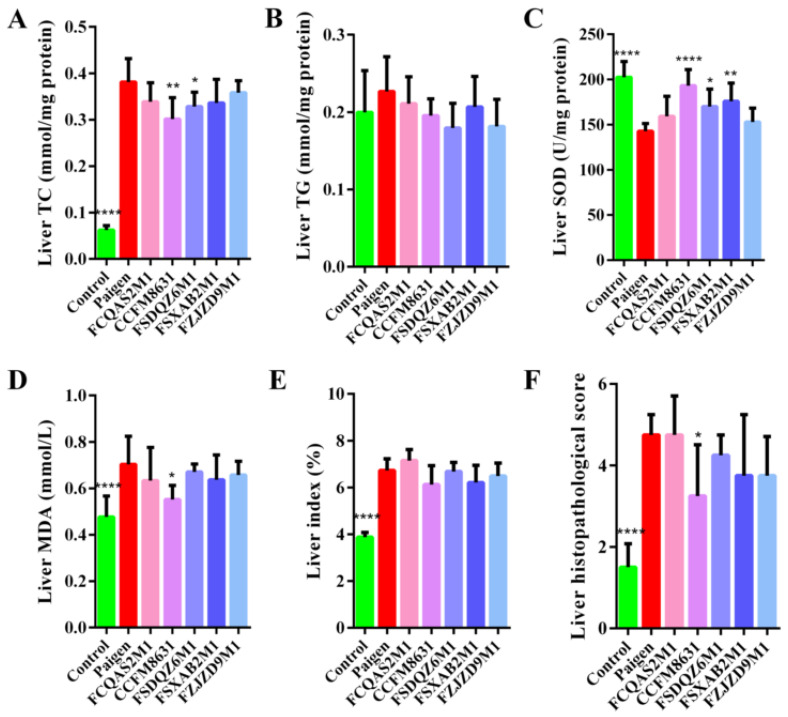
Analysis results of liver biochemical parameters and histopathological score. (**A**) TC. (**B**) TG. (**C**) SOD. (**D**) MDA. (**E**) Liver index (%). (**F**) Liver histopathological score. *n* = 8. Compared with Paigen group, * *p* < 0.05, ** *p* < 0.01, **** *p* < 0.0001.

As shown in Figure 2A, there was a significant difference in liver TC concentrations between the control and Paigen groups. Probiotic supplementation reduced liver TC concentrations to varying degrees, with only *L. reuteri* CCFM8631 and *L. casei* FSDQZ6M1 showing significant effects. As shown in Figure 2B, there was no significant difference in liver TG concentrations between the control and Paigen groups. Probiotic supplementation had a certain alleviating effect on the liver TG concentration, but this effect was not significant. As shown in Figure 2C,D, the Paigen atherogenic diet significantly affected SOD activity and MDA concentration in the liver. *L. reuteri* CCFM8631, *L. casei* FSDQZ6M1 and *B. breve* FSXAB2M1 significantly increased hepatic SOD activity in mice fed a Paigen atherogenic diet, while the other two strains had no significant effect. *L. reuteri* CCFM8631 significantly decreased MDA concentrations in the livers of mice fed a Paigen atherogenic diet, while the other four strains had no significant effect. As shown in Figure 2E, liver index values were significantly higher in the Paigen group than in the control group. Probiotic supplementation had different effects on liver index values, but there were no statistically significant effects. Although the levels of pro-inflammatory cytokines (TNF-α, IL-1β and IL-6) in Paigen atherogenic diet treatment were significantly higher than that in the control group, the supplementation of probiotics had no significant effects on those pro-inflammatory cytokines (Figure S2).

### 3.3. Lactobacillus Reuteri CCFM8631 Significantly Alleviated Liver Injury, but Only Slightly Decreased the Production of Foam Cells in the Abdominal Aorta and Aortic Arch after Paigen Atherogenic Diet Treatment

**Figure 3:**
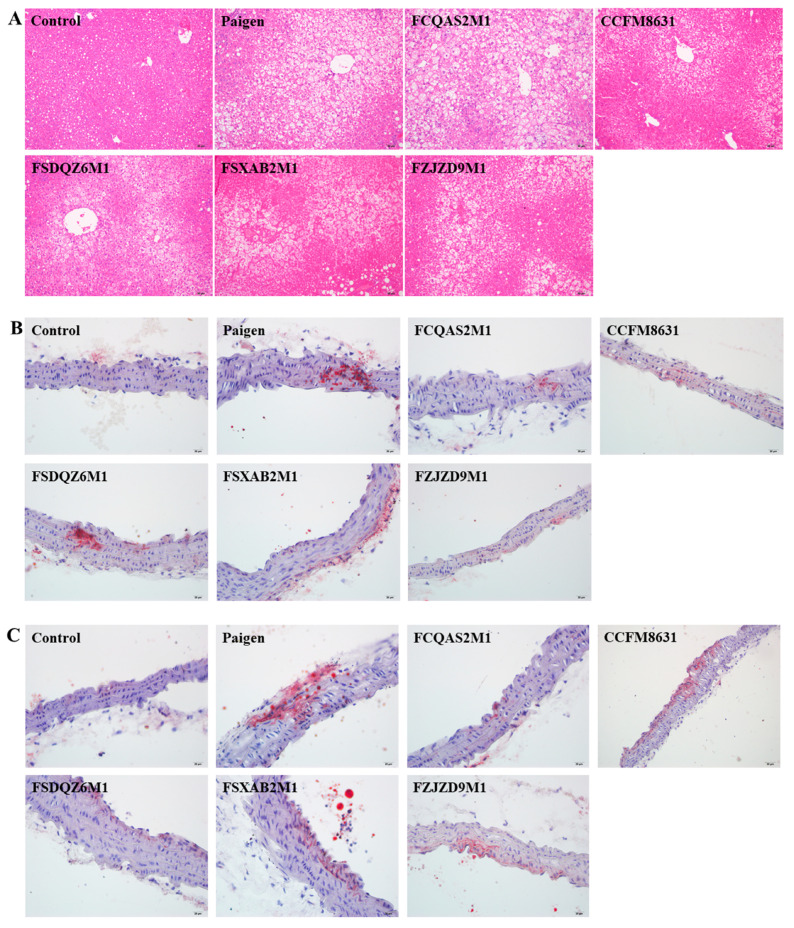
Histopathological analysis. (**A**) Liver HE staining. (**B**) Abdominal aorta oil red O staining. (**C**) Aortic arch oil red O staining. Specimens were photographed by light microscopy. (Magnification: ×200; HE staining, Scale bar: 50 μm; Oil red O stain, Scale bar: 20 μm).

HE staining was used to observe pathological changes in mouse liver tissue. As shown in Figure 2F and Figure 3A, compared with mice in the control group, those in the Paigen group showed a slightly disordered liver structure, with severe hepatocyte steatosis and slight focal inflammatory cell infiltration. The pathological changes observed in the probiotic intervention groups were generally similar to those observed in the Paigen group. However, the liver lesions were significantly less severe in mice supplemented with *L. reuteri* CCFM8631, and slightly, but not significantly, less severe in mice supplemented with *B. breve* FSXAB2M1 and *B. adolescentis* FZJZD9M1. *L. plantarum* FCQAS2M1 and *L. casei* FSDQZ6M1 did not alleviate the liver lesions. Vascular endothelial cells form foam cells due to lipid deposition and then, eventually, form plaques. Through Oil Red O staining of the abdominal aorta and aortic arch (Figure 3B,C), we observed obvious foam cells in the abdominal aorta and aortic arch after Paigen atherogenic diet treatment. Supplementation with *Lactobacillus* or *Bifidobacterium* alleviated foam cell formation to varying degrees.

### 3.4. Lactobacillus Reuteri CCFM8631 Significantly Affected the Composition of Short-Chain Fatty Acids in Mice Fed a Paigen Atherogenic Diet

**Figure 4:**
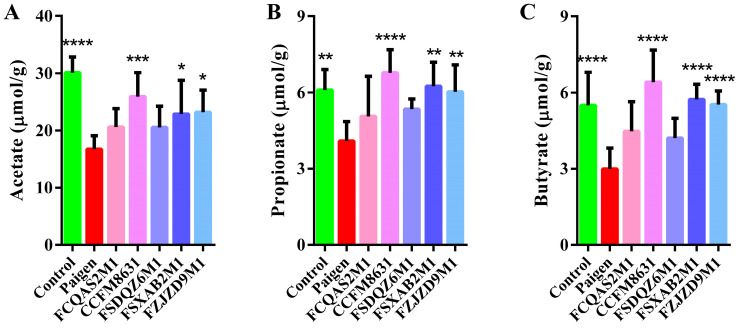
*Lactobacillus* and *Bifidobacterium* could improve the composition of faecal short-chain fatty acids to a certain extent. Levels of (**A**) acetate, (**B**) propionate, (**C**) butyrate. *n* = 8. Compared with Paigen group, * *p* < 0.05, ** *p* < 0.01, *** *p* < 0.001, **** *p* < 0.0001.

Acetate, propionate and butyrate are the three most abundant SCFAs in mouse faeces. As shown in Figure 4A–C, the faecal concentrations of acetate, propionate and butyrate were significantly lower in the Paigen group than in the control group. Probiotic supplementation regulated the faecal SCFA composition to varying degrees. *L. reuteri* CCFM8631, *B. breve* FSXAB2M1 and *B. adolescentis* FZJZD9M1 significantly increased the concentrations of acetate, propionate and butyrate.

### 3.5. Supplementation of Lactobacillus and Bifidobacterium Alleviated the Gut Microbiota Dysbiosis Caused by Paigen Atherogenic Diet Treatment

**Figure 5:**
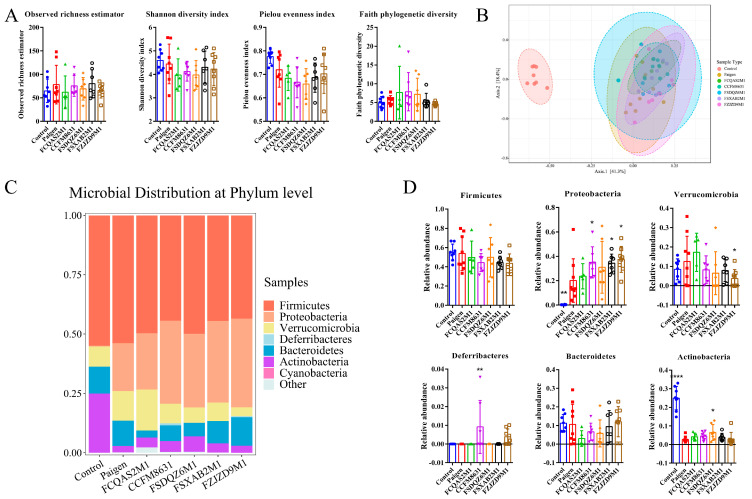
*Lactobacillus* and *Bifidobacterium* modulated the composition of intestinal flora. (**A**) α diversity of microbial faeces. (**B**) Principal coordinates analysis of microbial taxa. (**C**) Microbial distribution at phylum level. (**D**) Relative abundance of the phylum level. *n* = 8. Compared with Paigen group, * *p* < 0.05, ** *p* < 0.01, **** *p* < 0.0001.

To explore the effect of probiotic supplementation on the intestinal flora of mice fed a Paigen atherogenic diet, we performed 16S rDNA analysis of the mouse faeces samples. As seen in Figure 5A, α diversity analysis of mouse faecal flora showed no significant difference in the observed richness estimator, Shannon diversity index, Pielou evenness index or Faith’s Phylogenetic Diversity index values between the control and Paigen groups. As shown in Figure 5B, β diversity analysis showed a significant difference in the flora structure between the control and Paigen groups. Meanwhile, there was an overlap among the probiotic intervention groups and the Paigen group, indicating that there was no significant difference in the flora structure between these groups.

At the phylum level, Firmicutes showed the highest relative abundance in the control group, followed by Actinobacteria and Bacteroidetes (Figure 5C). In the Paigen group, Firmicutes showed the highest relative abundance, followed by Proteobacteria and Verrucomicrobia. The relative abundance of Proteobacteria was significantly higher, while that of Actinobacteria was significantly lower in the Paigen group compared with the control group (Figure 5D). Probiotic supplementation regulated the relative abundance of faecal microbes at the phylum level in mice fed a Paigen atherogenic diet. The relative abundance of Deferribacteres was very low and barely detectable in both the control and Paigen groups (Figure 5C), but it was higher in mice treated with *L. reuteri* CCFM8631 or *B. adolescentis* FZJZD9M1. Moreover, the relative abundance of Deferribacteres was significantly higher in the CCFM8631 group compared with the Paigen group (Figure 5D).

**Figure 6:**
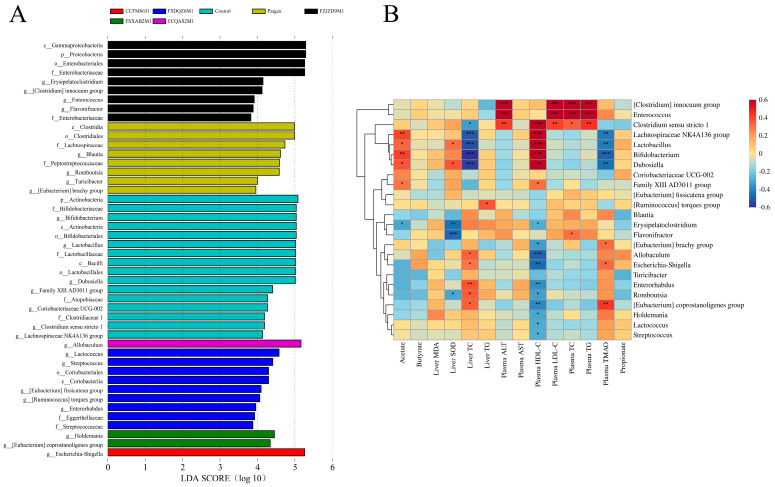
*Lactobacillus* and *Bifidobacterium* could remodel the structure of the flora microbiota. (**A**) Plot LEfSe Results of microbial faeces. LDA > 2, *p* < 0.05 (**B**) Correlation between measured indicators and genus with significant differences. * *p* < 0.05, ** *p* < 0.01, *** *p* < 0.001.

Linear discriminant analysis effect size analysis of mouse faecal flora was performed using the Galaxy tool (http://huttenhower.sph.harvard.edu/galaxy/) (accessed on 1 March 2022), and the genera with significant differences were selected for Pearson’s correlation analysis with the measured indicators. As shown in Figure 6A,B, the abundance of some genera was closely related to the measured indicators. The relative abundance of the genera that played a significant role is shown in Figure S3. Among them, the abundance of the *Clostridium innocuum* group and *Enterococcus* was significantly positively correlated with plasma ALT, LDL-C, TC and TG concentrations. The relative abundance of the *C. innocuum* group and *Enterococcus* was slightly, but not significantly, higher in the Paigen group than in the control group. The abundance of *Clostridium sensu stricto 1*, the *Lachnospiraceae* NK4A136 group, *Lactobacillus*, *Bifidobacterium* and *Dubosiella* was significantly positively correlated with faecal acetate and plasma HDL-C concentrations and negatively correlated with hepatic TC and plasma TMAO concentrations. The abundance of these genera decreased significantly in the Paigen group compared with the control group, and probiotic supplementation regulated their relative abundance to varying degrees. For example, supplementation with *B. adolescentis* FZJZD9M1 increased the relative abundance of *Clostridium sensu stricto 1*, *Bifidobacterium* and *Dubosiella* and supplementation with *L. reuteri* CCFM8631 increased the relative abundance of the *Lachnospiraceae* NK4A136 group, *Lactobacillus* and *Dubosiella*. The abundance of *Erysipelatoclostridium*, *Flavonifractor* and *Romboutsia* was negatively correlated with faecal acetate concentration, hepatic SOD activity and plasma HDL-C concentration. The abundance of these genera increased significantly in the Paigen group compared with the control group, and probiotic supplementation regulated their relative abundance to varying degrees. *L. plantarum* FCQAS2M1, *L. reuteri* CCFM8631 and *B. breve* FSXAB2M1 decreased the relative abundance of *Erysipelatoclostridium* and significantly decreased the relative abundance of *Romboutsia*. The abundance of *Allobaculum*, *Escherichia-Shigella*, *Enterorhabdus* and the *Eubacterium coprostanogenes* group was positively correlated with hepatic TC and plasma TMAO concentrations and negatively correlated with plasma HDL-C concentration. The abundance of these genera was significantly increased in the Paigen group compared with the control group, and *L. reuteri* CCFM8631 significantly decreased the relative abundance of *Enterorhabdus*.

**Figure 7:**
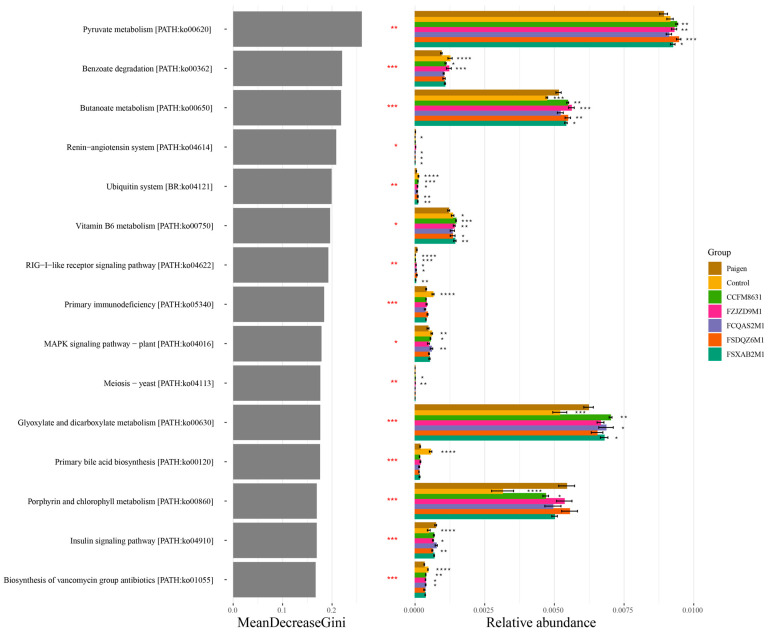
*Lactobacillus* and *Bifidobacterium* could regulate the potential function of the gut microbiota. Top 15 pathways/functions by microeco. *n* = 8. Compared with Paigen group, * *p* < 0.05, ** *p* < 0.01, *** *p* < 0.001, **** *p* < 0.0001.

We used picrust2 (Phylogenetic Investigation of Communities by Reconstruction of Unobserved States) to predict the function of faecal flora components, and used microeco [19] to identify the top 15 pathways/functions based on random forests and relative abundance data. The key pathways/functions and their significance are shown in Figure 7, Pearson’s correlation analysis of the measured indicators was also performed, as shown in Figure S4. Butanoate metabolism, porphyrin and chlorophyll metabolism, glyoxylate and dicarboxylate metabolism, the insulin signalling pathway, the renin–angiotensin system and the retinoic acid-inducible gene I (RIG-I)-like receptor signalling pathway were negatively correlated with faecal acetate concentration, hepatic SOD activity and plasma HDL-C concentration, but positively correlated with hepatic TC and plasma ALT and TMAO concentrations. These pathways were significantly activated in the Paigen group compared with the control group, and probiotic intervention regulated these pathways to varying degrees. For example, *L. reuteri* CCFM8631 significantly inhibited porphyrin and chlorophyll metabolism, the RIG-I-like receptor signalling pathway, the insulin signalling pathway and the renin–angiotensin system. However, probiotic intervention activated butanoate metabolism and glyoxylate and dicarboxylate metabolism in mice fed a Paigen atherogenic diet. Primary bile acid biosynthesis, biosynthesis of vancomycin group antibiotics, primary immunodeficiency, benzoate degradation and the ubiquitin system were positively correlated with faecal acetate concentration, hepatic SOD activity, plasma HDL-C concentration and faecal propionate concentration, but negatively correlated with hepatic TC and plasma TMAO concentrations. These pathways were significantly inhibited in the Paigen group compared with the control group, and probiotic intervention regulated these pathways to varying degrees. *L. reuteri* CCFM8631 significantly activated benzoate degradation and the ubiquitin system. In addition, pyruvate metabolism and vitamin B6 metabolism were inhibited in the Paigen group compared with the control group, but probiotic supplementation rescued this inhibition, and *L. reuteri* CCFM8631 significantly activated these pathways.

## 4. Discussion

Diets with high fat and cholesterol content are associated with an increased risk of obesity and metabolic diseases in mice and humans. Many previous studies have demonstrated a close relationship between the intestinal flora and health and disease. Many diseases are associated with intestinal flora dysbiosis. For example, TMAO, an intestinal metabolite produced as a result of choline metabolism by intestinal microorganisms, promotes the occurrence of CVD [20]. Some probiotics have been reported to play an important role in the prevention of diseases, such as metabolic syndrome [21], diabetes [22], non-alcoholic fatty liver disease [23] and colitis [24]. In this study, *L. reuteri* CCFM8631 was found to alleviate hypercholesterolaemia induced by a Paigen atherogenic diet.

High bacterial richness and diversity, which reflects the stability and resilience of the ecosystem, are generally considered to be the main characteristics of a healthy intestinal microbial ecosystem. Disease has been proved to be correlated with gut bacterial richness and diversity through faecal microbiome analysis [25,26]. In this study, although no significant effects on bacterial richness and diversity were caused, probiotics supplementation significantly affected gut microbiota structure. Intestinal microorganisms produce or regulate microbial metabolites. There is increasing evidence showing that disorders of the composition and function of intestinal microbes play important roles in the development of diseases related to metabolism [27,28]. The majority (more than 95%) of gut microbes in mice and humans belong to the phylum Firmicutes and the phylum Bacteroidetes [29]. In our study, Firmicutes showed the highest relative abundance in both the control and Paigen groups and Deferribacteres species were barely detectable. However, after supplementation with probiotics, especially *L. reuteri* CCFM8631 and *B. adolescentis* FZJZD9M1, the relative abundance of Deferribacteres significantly increased. Deferrobacteria are obligate or facultative anaerobes. They maintain iron balance in the intestine. Abnormal iron metabolism increases the risk of disease and promotes tumour growth. Jiao et al. [30] found that blueberry polyphenols, such as orlistat, increase the relative abundance of Deferribacteres in the intestine under high-fat diet conditions. Supplementation with *Codonopsis foetens* increases the relative abundance of intestinal Deferribacteres and alleviates the symptoms of intestinal cancer induced by 1,2-dimethylhydrazine [31]. Thus, *L. reuteri* CCFM8631 and *B. adolescentis* FZJZD9M1 have the potential to maintain the iron balance of the intestines and prevent disease development.

Recent studies in mice and humans have shown that dyslipidaemia is related to changes in the intestinal microecology, such as a decrease in microbial diversity and a decrease in the abundance of Bacteroidetes [32]. In addition, compared with conventionally raised mice, germ-free mice have a reduced incidence of obesity and decreased hepatic TG concentrations and are more resistant to diet-induced obesity [33,34]. Population-based cohort analyses have also shown that obesity, related metabolic disorders and dyslipidaemia are correlated with the intestinal bacterial taxa. The correlation between intestinal bacterial taxa and blood lipid concentrations may be mediated by body mass index or other metabolic disorders [35,36]. In our study, mice fed a Paigen atherogenic diet showed excessively high lipid and cholesterol concentrations. Plasma TC, TG, LDL and TMAO concentrations and hepatic TC and TG concentrations increased significantly, and plasma HDL-C concentration decreased significantly in the Paigen group compared with the control group. Pearson’s correlation analysis showed that the abundance of certain bacteria was related to metabolic function. After supplementing with probiotics, the structure of the intestinal flora improved, and the probiotics reversed the changes in the intestinal flora caused by the Paigen atherogenic diet. *Lactobacillus* species in the intestine reduce inflammation, improve intestinal immune activity, synthesise bile salt hydrolase, activate the farnesoid X receptor signalling pathway and hepatic lipid metabolism, promote the metabolism of blood lipids and cholesterol and maintain host health [37]. In our study, as expected, *L. reuteri* CCFM8631 supplementation significantly increased the relative abundance of *Lactobacillus*, which played an important role in reducing inflammation and promoting lipid metabolism. Vitamin B6 reduces homocysteine levels, which helps decrease the risk of developing atherosclerosis [38]. We found that *L. reuteri* CCFM8631 activated vitamin B6 metabolism, indicating a potential role in reducing atherosclerotic CVD. Previous studies have shown that an increase in the abundance of the *Lachnospiraceae* NK4A136 group in mouse faeces is associated with decreased glucose concentrations, improved glucose tolerance, and reduced inflammation [39]. We also found that *Lachnospiraceae* NK4A136 group supplementation was correlated with increased plasma HDL-C concentration and decreased plasma TMAO and liver TC concentrations. Galié et al. [40] also found that a Mediterranean diet increases the relative abundance of the *Lachnospiraceae* NK4A136 group in faeces, reduces insulin resistance and decreases the risk of cardiometabolic diseases.

SCFAs produced by intestinal fermentation are mainly produced in the proximal colon. *Bacteroides*, which are butyrate-producing bacteria, and *Clostridium* play important roles in the fermentative production of SCFAs [41], which are then absorbed into the portal vein to participate in host metabolism. SCFAs reduce the pH of the intestinal cavity; inhibit the growth of pathogenic microorganisms; improve the absorption of nutrients; provide energy for intestinal epithelial cells [42]; participate in lipid metabolism, glucose homeostasis, intestinal inflammation and neurogenesis; regulate the host immune system and maintain the homeostasis of the intestinal immune system [43]. We found that acetate, propionate and butyrate concentrations were significantly reduced in the Paigen group compared with the control group. Probiotic supplementation adjusted the composition of SCFAs. Pearson’s correlation analysis showed that the abundance of certain bacteria was related to SCFA concentration. The *Lachnospiraceae* NK4A136 group includes butyrate-producing bacteria that maintain the integrity of the mouse intestinal barrier and their abundance is negatively correlated with intestinal permeability [44]. Butyrate is one of the main SCFAs produced by the intestinal flora. It enhances the integrity of the epithelial barrier and inhibits inflammation. Thus, butyrate plays an important role in maintaining gastrointestinal health [45]. We found that a Paigen atherogenic diet reduced the relative abundance of the *Lachnospiraceae* NK4A136 group and butanoate metabolism, whereas *L. reuteri* CCFM8631 supplementation increased the relative abundance of *Lachnospiraceae* NK4A136 group in the faeces, activated butanoate metabolism and increased the concentration of SCFAs. These effects are beneficial as they enhance the integrity of the intestinal barrier and maintain intestinal health. *Romboutsia* species are also associated with the production of SCFAs [46]. Pearson’s correlation analysis showed that the abundance of *Romboutsia* was negatively correlated with acetate, propionate and butyrate concentrations. The Paigen atherogenic diet significantly increased the relative abundance of *Romboutsia* in faeces, but *L. reuteri* CCFM8631, *B. breve* FSXAB2M1 and *B. adolescentis* FZJZD9M1 supplementation significantly decreased the relative abundance of *Romboutsia* in faeces and significantly increased the concentrations of acetate, propionate and butyrate. These three strains also significantly decreased plasma TC and LDL-C concentrations and alleviated liver injury. Increasing the concentration of SCFAs plays an important role in alleviating hypercholesterolaemia.

## 5. Conclusions

In conclusion, the Paigen atherogenic diet has a high cholesterol and TG content, which increases the risk of developing atherosclerosis. Decreasing the serum cholesterol and TG concentrations is considered to be an effective strategy to prevent atherosclerotic CVD. *L. reuteri* CCFM8631 may alleviate the symptoms of hypercholesterolaemia by decreasing blood lipid concentrations; increasing faecal SCFA concentrations (acetate, propionate and butyrate); improving the structure of the gut microbiota, especially by increasing the relative abundance of Deferribacteres, the *Lachnospiraceae* NK4A136 group, *Lactobacillus* and *Dubosiella* and decreasing the relative abundance of *Erysipelatoclostridium* and *Romboutsia*; and activating butanoate metabolism and vitamin B6 metabolism. These effects of *L. reuteri* CCFM8631 play an important role in the prevention of atherosclerotic CVD.

## Figures and Tables

**Table 1:** *Lactobacillus* and *Bifidobacteria* used in this study.

Serial Number	Species	Original Number	Sample
1	*Lactobacillus plantarum*	FCQAS2M1	Human faeces
2	*Lactobacillus reuteri*	CCFM8631	Human faeces
3	*Lactobacillus casei*	FSDQZ6M1	Human faeces
4	*Bifidobacterium breve*	FSXAB2M1	Human faeces
5	*Bifidobacterium adolescentis*	FZJZD9M1	Human faeces

## Supplementary Materials

The following supporting information can be downloaded at: https://www.mdpi.com/article/10.3390/nu14061272/s1, Figure S1: The effects of *Lactobacillus* and *Bifidobacterium* administration on body weight, food intake and water intake; Figure S2: Effects of *Lactobacillus* and *Bifidobacterium* on the levels of liver pro-inflammatory cytokines; Figure S3: Relative abundance of the genus level. *n* = 8. Compared with Paigen group; Figure S4: Correlation between the measured indicators and the significant pathway/function.
