# Supplementary material for: Lactobacillus reuteri CCFM8631 Alleviates Hypercholesterolaemia Caused by the Paigen Atherogenic Diet by Regulating the Gut Microbiota
Source: Nutrients. 2022 Mar 17;14(6):1272. doi: 10.3390/nu14061272 (PMC8953203; doi:10.3390/nu14061272)
Supplement: Supplementary file 1 [file nutrients-14-01272-s001.zip › nutrients-1610229-supplementary.pdf]

Supplementary Materials and Methods

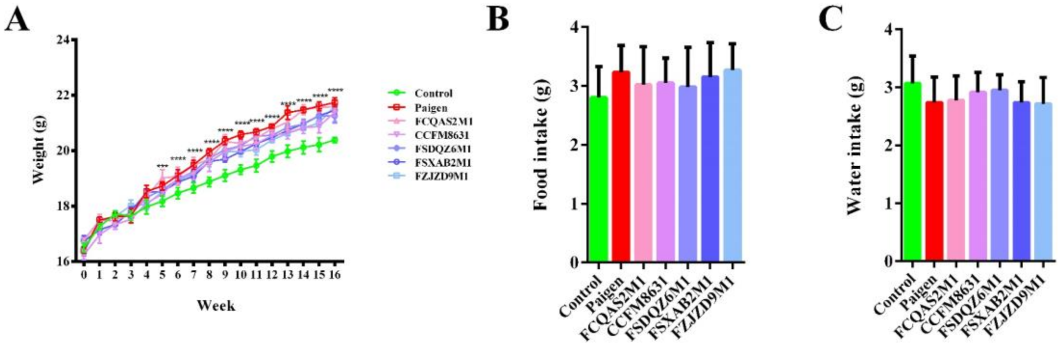

Figure S1. The effects of *Lactobacillus* and *Bifidobacterium* administration on body weight, food intake and water intake. (A) Effects of *Lactobacillus* and *Bifidobacterium* on body weight. Compared with Control group, *** $p < 0.001$, **** $p < 0.0001$. (B) Effects of *Lactobacillus* and *Bifidobacterium* on food intake. (C) Effects of *Lactobacillus* and *Bifidobacterium* on water intake. n=8.

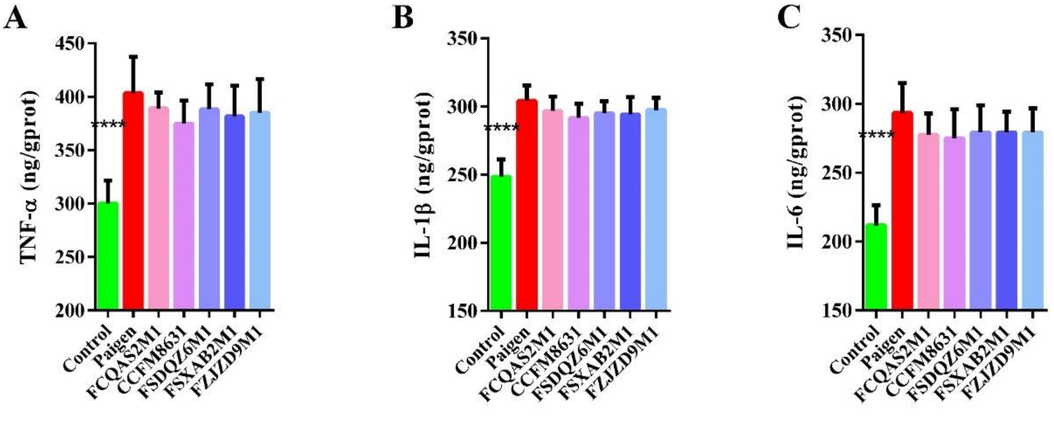

Figure S2. Effects of *Lactobacillus* and *Bifidobacterium* on the levels of liver pro-inflammatory cytokines. (A) Liver TNF-α concentrations. (B) Liver IL-1β concentrations. (C) Liver IL-6 concentrations. Compared with Paigen group, **** $p < 0.0001$. n=8.

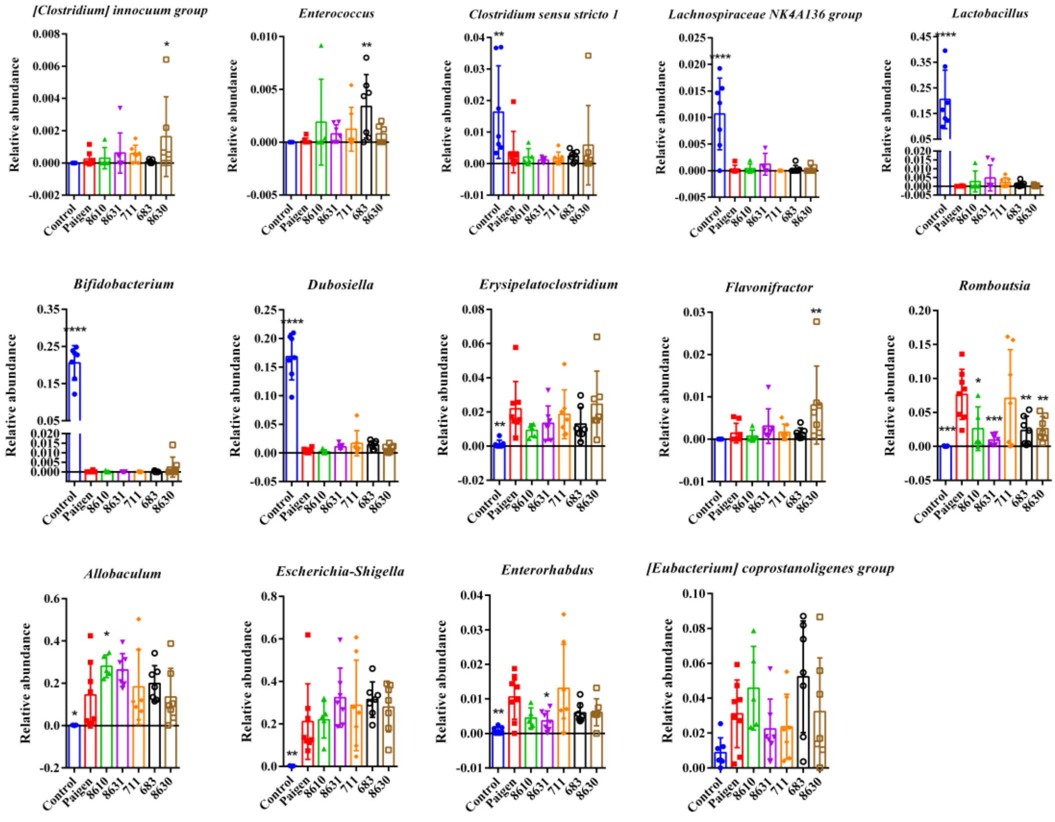

Figure S3. Relative abundance of the genus level. n=8. Compared with Paigen group, * $p < 0.05$, ** $p < 0.01$, *** $p < 0.001$, **** $p < 0.0001$.

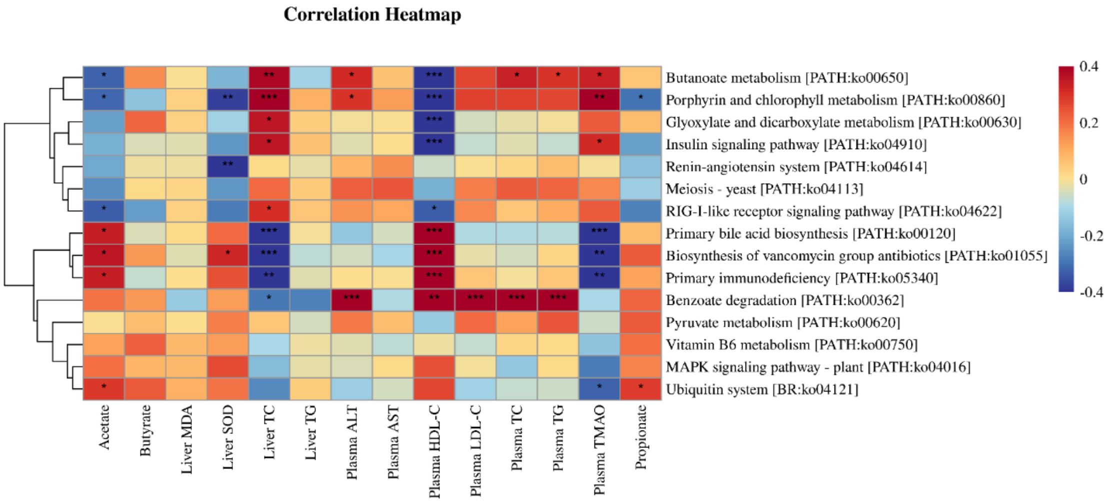

Figure S4. Correlation between the measured indicators and the significant pathway/function. * $p < 0.05$, ** $p < 0.01$, *** $p < 0.001$.
